# Impact of breast milk on cortical pain response in newborns during the heel prick procedure: a randomized controlled trial

**DOI:** 10.1038/s41372-024-02081-4

**Published:** 2024-08-12

**Authors:** Ozgul Bulut, Seyma Cagla Topaloglu, Nurgul Bulut, Meryem Hocaoglu, Sertac Arslanoglu

**Affiliations:** 1 Department of Pediatrics, Division of Neonatology, Istanbul Medeniyet University Goztepe Prof. Dr. Suleyman Yalcın City Hospital, Istanbul, Turkey; 2 Department of Biostatistics and Medical Informatics, Istanbul Medeniyet University Goztepe Prof. Dr. Suleyman Yalcın City Hospital, Istanbul, Turkey; 3 Department of Obstetrics and Gynecology, Istanbul Medeniyet University Goztepe Prof. Dr. Suleyman Yalcın City Hospital, Istanbul, Turkey

**Keywords:** Medical research, Outcomes research

## Abstract

**Objective:**

To investigate the effects of breast milk on cortical pain response and behavioral response in newborns during heel-prick procedure.

**Study design:**

A prospective, randomized controlled trial was conducted on healty-term newborns, undergoing heel blood sampling. Infants were randomly assigned to study group with receive orally 2 ml breast milk (*n* = 45) or a control group with no intervention (*n* = 45). A near-infrared spectroscopy device was used to monitor regional cerebral oxygen saturation (rScO_2_), while neonatal pain expression was assessed by Neonatal Pain, Agitation, and Sedation Scale (N-PASS).

**Results:**

The N-PASS score (*p* = 0.001) and the crying time (*p* = 0.017) were significantly lower in the study group compared to the control group. Although the mean rScO_2_ values decreased in both groups during the procedure, the percent change in rScO_2_ was not significant difference between two groups.

**Conclusion:**

Breast milk administration decreases behavioral responses to a noxious stimulus without reducing the cortical response to pain.

**Clinical trial registration:**

This trial was registered under ClinicalTrials.gov identifier no. NCT05961904.

## Introduction

During the first few days of life, newborns are exposed to painful and stressful procedures [1, 2]. One of these is the metabolic disease screening test using the heel-prick procedures [3, 4]. Newborn screening is crucial to detect several congenital genetic and metabolic disorders at an early stage for the earliest possible recognition and management of affected newborns and to prevent morbidity, mortality, and disabilities associated with inherited metabolic disorders [5]. Pain induced by these procedures is ineffectively prevented or inadequately treated^1^. Consequently, this may have short- and long-term negative effects on the pain response and neurodevelopmental outcomes [6–8]. Thus, effectively identifying, assessing, and managing neonatal pain are crucial to minimizing its impact on the intermediate- and long-term outcomes in newborns [2, 9].

Considering that infants are unable to communicate with their caregivers, assessing neonatal pain is challenging [10]. However, when neonates respond to painful stimuli, biochemical, physiological, and behavioral changes occur [11, 12]. Thus, the most commonly used method to assess clinical pain is via behavioral and physiological surrogate measures of infant pain [8, 9]. However, neonatal staff report difficulty in assessing pain based on behavioral and physiological indicators, and several factors, such as gestational age, sex, prior pain exposure, illness, neurobehavioral state, and stress level, may influence these indicators [8, 13]. Therefore, the validity and reliability of these methods are questionable [14]. These observations emphasize the importance of considering a comprehensive multimodal approach for pain assessment to best estimate an infant’s pain [15]. More recently, changes in electrophysiological and hemodynamic brain activity in infants have been identified and characterized in response to noxious stimulation [8, 16]. As pain perception manifests in the brain, brain-derived approaches may provide the best surrogate measures of infant pain in the absence of language and may be further directly linked to the pain experience. Thus, simultaneously measuring the changes evoked in behavior, physiology, and the cortex following noxious events might provide the best approach to understanding the neonate’s pain experience [17, 18]. Near-infrared spectroscopy (NIRS) is a noninvasive technique widely used in neonatal pain research to measure functional activation of the cortex [19–21]. NIRS has revealed that blood sampling can activate the neonatal somatosensory [19], motor [22], and prefrontal [20] areas starting at the 25th week of postmenstrual age. Pain increases oxygen consumption with changes occurring in the primary somatosensory cortex on the brain surface [23]. Additionally, the relationship between the total Premature Infant Pain Profile (PIPP) score and hemodynamic response has been evaluated. A strong correlation between the magnitude of the hemodynamic response and changes in facial expression has been shown [24]. Thus, NIRS may be an excellent tool to observe these changes [18, 21], thus enhancing the quality of routine care and reducing neonatal stress.

During minor painful procedures in neonatal units, nonpharmacological analgesia is commonly used [10, 25, 26]. Among the analgesics studied for neonatal pain, breast milk is a natural substance beneficial and nutritious for infants [27]. Breast milk is composed of lactose (which has a sweet taste) and tryptophan (which may facilitate endogenous opioid secretion to buffer biobehavioral pain responses) [28]. Several systematic reviews and meta-analyses have described the effectiveness of breastfeeding or breast milk in decreasing neonatal pain from both behavioral (e.g., crying, facial expression, and fussiness) and physiological (e.g., heart rate) perspectives during painful procedures [10, 29]. However, in addition to evaluating pain using the usual pain scales, the effectiveness of nonpharmacological analgesia methods used to prevent pain has recently been questioned. NIRS was the first brain imaging method used to investigate the effect of sucrose on brain activity following heel lancing [30] with no significant difference reported in cerebral blood volume between the sucrose and placebo groups despite reduced heart rate and crying after sucrose administration. Therefore, investigating cortical hemodynamic responses to painful stimuli in neonates using nonpharmacological methods can substantially increase our understanding of pain management in infants. Whether breast milk administration alters cortical brain activation in neonates is presently unclear. Therefore, we conducted a trial to assess whether breast milk can relieve cortical pain during a painful metabolic disease screening procedure.

## Methods

### Study design and participants

**Fig. 1:**
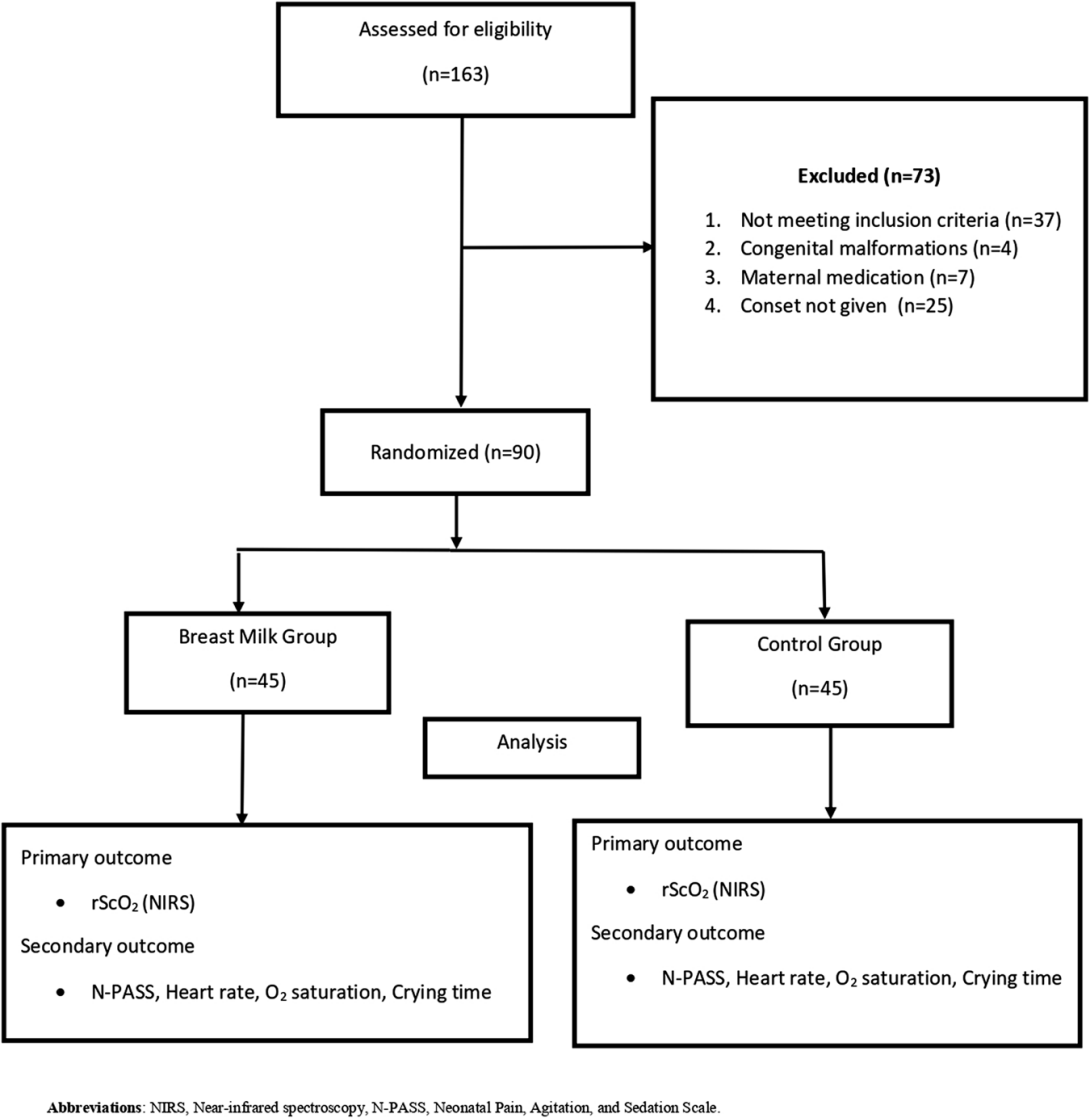
Flow diagram of the study.

A prospective, randomized controlled trial was conducted in a tertiary care Istanbul Medeniyet University Goztepe Prof. Dr. Suleyman Yalcın City Hospital from June and August 2021. Healthy, full-term newborn infants who were followed up at the maternity ward were assessed for inclusion before heel-prick procedure for routine newborn screening on post-natal day one or two (Fig. 1). This study was approved by the local Ethics Committee (2021/0312). Written informed consent was obtained from the parents before enrollment.

The inclusion criteria selected neonates born after 37 weeks of gestation, postnatal age more than 24 h (h), absence of congenital malformations, ongoing intubation, and mechanical ventilation, who had not received analgesic, anesthetic, or sedative drugs. None of the newborns had previously experienced any painful procedure.

### Procedure

Newborn infants were randomly assigned to one of the two groups to receive either expressed breast milk (study group) or a control group with no intervention by means of cards in sequentially numbered sealed opaque envelopes. Expressed breast milk was collected for all the babies from their mothers in a sterile container before the procedure. The breast milk was administered directly into the anterior surface of the tongue with a 2 mL syringe 2 min before the heel prick was done on the examination table. A trained neonatal nurse administered all breast milk.

### Near-infrared spectroscopy (NIRS) recording

**Fig. 2:**
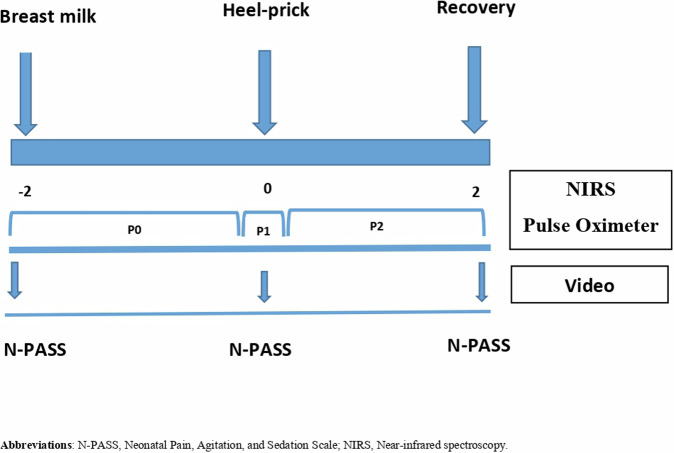
Timeline for measuring the outcome of this study.

In this study, a single channel near-infrared spectrophotometer (INVOS^TM^ 5100c Covidien Medtronic; Mansfield CA, USA) was used to measure changes in regional cerebral oxygen saturation (rScO_2_). The technique depends on the transparency of biological tissue to near-infrared light and uses the fact that the absorption of near-infrared light by oxygenated and deoxygenated hemoglobin depends on the oxygenation state [31, 32], therefore, tissue oxygen consumption can be continuously observed by monitoring rScO_2_ [33]. The NIRS probe was placed on the center of the forehead and a pulse oximeter probe (Covidien-Nellcor, USA) was applied to the infant’s right hand to measure heart rate (HR) and peripheral oxygen saturation (SpO_2_). The rScO_2_ data was sampled every 5.0 s and exported to a personal computer via digital output during the entire study period. The multidimensional assessment was recorded, starting 2 min before the heel prick (P0 = Baseline), continuing throughout the procedure (P1 = Painful procedure), and ending 2 min after the heel prick (P2 = Recovery) (Fig. 2). The average of the values measured in this three periods was calculated for each infant. In addition, the percentage change in rScO_2_, HR and SpO_2_ was determined. The difference between the mean values during and after the procedure and the baseline mean values were divided by the baseline mean value and was multiplied by 100 for calculation of the percentage change.

### Pain assessment

Pain was assessed using the N-PASS scale. Hummel et al. demonstrated high inter-rater reliability, internal consistency and test-retest reliability of the N-PASS. In the ‑PASS tool, five indicators are included as follows: crying/irritability, behavior/state, facial expression, extremities/tone, and vital signs (heart rate, respiratory rate, blood pressure, and/or oxygen saturation). Each of the five criteria are graded from −2 to +2. A score >+3 indicates pain, and a score of >−3 indicates sedation. Scores for sedation graded 0, −1 and −2 were not used as sedated infants were excluded from this study. As for preterm infants, this system adjusts the pain score in order to approximate the response of term infants by adding points to the calculated result on the basis of gestational age [34]. The N-PASS was not coded continuously. It was scored 2 min before, the time of, and 2 min after heal prick for each infant (Fig. 2).

Crying time was defined as the duration of crying within the five minutes of recording time while the duration of the first cry was defined as audible distressed vocalizations with a continuous pattern before a quiet interval of five seconds immediately after the painful stimulus.

### Heel-prick protocol

During the study, all the babies were taken into a baby room for heel prick and were laid supine on the examination table. The babies were wearing only diapers and bodysuits. Blood samples were collected using a 21-gauge needle device on the foot (the outer right side of the ball) following a standardized procedure and all heel pricks were performed by the same nurse who is experienced in blood sampling. The heel prick was initiated at the point in time when the nurse touched the heel of the neonate, and the duration ended when her hands left the heel. Data were collected within 30 s of the painful stimulation. All infants were awake and stable condition at the time of the procedure. The environment around the infant during the study was kept as quiet as possible, avoiding any kind of visual, auditory, and olfactory external stimuli. To avoid confounding by other pain-relieving methods, it was ensured that non-nutritive sucking, touching or swaddling of the baby was not done during the procedure.

All infants were video recorded continuously 2 min before until 2 min after the procedure using a digital camera. The camera was positioned to provide a clear view of the procedure, the infant’s face and body, the pulse oximeter displaying SpO_2_ and HR, and the NIRS monitor displaying rScO_2_. The crying time and N-PASS scores were analyzed by two researchers from the video records of the procedure, who were blind to the study.

### Outcomes

The primary outcome measure was the effect of breast milk on cortical response to pain by one heel prick, recorded with NIRS. The secondary outcome was the effect of breast milk on behavioral pain response using the N-PASS score, vital signs and crying time.

### Data analysis

Statistical analysis was performed using IBM SPSS (Statistical Package for the Social Sciences), version 22.0 software (SPSS Inc., Chicago, IL). GraphPad Prism 10.2.3 software was used for figure. The sample size was calculated using the pain responses measured by the N-PASS. Using data from previous studies an increase of two points in N-PASS scores during the painful procedure was considered clinically significant [35]. The minimum sample size was calculated as 44 infants per group to permit a power of %95 with an α of 0.05. Descriptive statistics were presented as mean, SD, median (interquartile range), frequency, and percentage. The distribution characteristics of continuous variables were evaluated with Shapiro-Wilk the test since the sample size was less than 50 in each group. The Student t-test was used for parametric data and the Mann-Whitney U test was used for nonparametric data where appropriate. The Pearson chi-square (χ2) test was used to compare categorical variables as appropriate. In additon, the Repeated Measure ANOVA test was used to evaluate the percent changes of the parameters measured during and after the procedure in both groups compared to baseline values, both in terms of time and groups. Multiple comparisons of significant results were evaluated with Bonferroni correction. The correlation between variables was analyzed using the Spearman ρ correlation test. A probability (P) value < 0.05 was considered statistically significant.

## Results

Ninety term newborns, including the study group (*n* = 45) and control group (*n* = 45), were included in this study. There were no significant differences in the demographic characteristics of the newborns between the two groups (*P* > 0.05) and are shown in Table 1.

**Table 1:** The comparison of the demographic characteristics of newborns between the groups.

	Study Group [*n* = 45]	Control Group [*n* = 45]	*p*
Gestational age (weeks)	39.3 ± 1.09 39 [37.7–42.3]	39.04 ± 1.18 39 [37.7–42.3]	0.201**
Birth weight (g)	3283 ± 440 3310 [2395–4440]	3352 ± 333 3340 [2270–3960]	0.405*
Male, *n* (%)	27 [60]	27 [60]	0.999***
Apgar score at 1 min	7.51 ± 0.99 8 [5–9]	7.56 ± 1.08 8 [5–10]	0.96**
Apgar score at 5 min	9.42 ± 0.72 10 [8–10]	9.64 ± 0.69 10 [8–10]	0.115**
Cesarean delivery, *n* (%)	18 [40]	26 [58]	0.092***
Postnatal age (day)	2.44 ± 0.5 2 [2–3]	2.56 ± 0.5 2 [1–2]	0.295**

^*^Student sample *t*-test **Mann Whitney- *U* test *** Pearson Ki-Kare testi

Data are shown as mean ± SD, median [min-max], or *n* (%).

### At baseline (before the procedure)

the comparison of basal vital signs (HR and SpO_2_), N-PASS scores, and rScO_2_ values did not show any statistical significance between the groups (*p* > 0.05) (Table 2).

**Table 2:** The comparison of the measurements between the groups during the study period.

Parameters	Study Group [*n* = 45]	Control Group [*n* = 45]	*p*
**Heart rate**			
Baseline	135.8 ± 13.5 136 [106–163]	139 ± 17.3 139 [99–171]	0.294*
During puncture	161.1 ± 17.5 157 [131–204]	163.5 ± 19.7 164 [99–202]	0.554**
After puncture	152.3 ± 21.3 151 [118-192]	161 ± 25.5 160 [99-219]	0.096*
**SpO**_**2**_ **(%)**			
Baseline	95.8 ± 2.8 96 [90–100]	95.1 ± 3.8 96 [86–100]	0.313*
During procedure	88.1 ± 5.8 89 [73–98]	87.7 ± 6.5 90 [74–98]	0.84**
After procedure	91.96 ± 5.8 94 [75–100]	91.4 ± 5.5 92 [72–100]	0.425**
**N-PASS**			
Baseline	0.27 ± 0.447 0 [0-1]	0.33 ± 0.674 0 [0–3]	0.962**
During procedure	7.24 ± 1.99 8 [2-10]	8.67 ± 1.128 9 [5–10]	0.001**
After procedure	1.36 ± 1.58 1 [0–5]	3.73 ± 3.26 3 [0–10]	0.001**
**rScO**_**2**_ **(%)**			
Baseline	82 ± 5.35 82 [70–92.3]	79.5 ± 7 79 [66–94]	0.052*
During procedure	79 ± 6 79 [68–93.3]	78 ± 6.8 77 [66–95]	0.385*
After procedure	78.1 ± 6.6 79 [60.5–92]	78.1 ± 7.2 77.4 [66–95]	0.987*
**Crying time (seconds)**	69.38 ± 33.16 71 [0–156]	86.16 ± 32.56 82 [33–173]	0.017*

^*^Student sample *t*-test, ** Mann Whitney- *U* test.

SpO_2_: Transcutaneous oxygen saturation.

N-PASS; Neonatal Pain, Agitation, and Sedation Scale.

rScO_2_ (%); Regional cerebral oxygen saturation.

### During the procedure

while there were a significant reduction in the SpO_2_ and an increase in the HR in both groups, there were no significant differences in the HR and the SpO_2_ between the groups (*p* > 0.05). The rScO_2_ values were 79 ± 6% in the study group and 78 ± 6.8% in the control group, with no significant difference between groups (*P* > 0.05). The N-PASS score was significantly lower in the study group than in the control group (7.24 ± 1.99 vs. 8.67 ± 1.128, *p* = 0.001, respectively). The crying time was significantly lower in the study group than in the control group (69.38 ± 33.16 sec vs. 86.16 ± 32.56 sec, *p* = 0.017, respectively) (Table 2).

### At recovery (after the procedure)

**Fig. 3:**
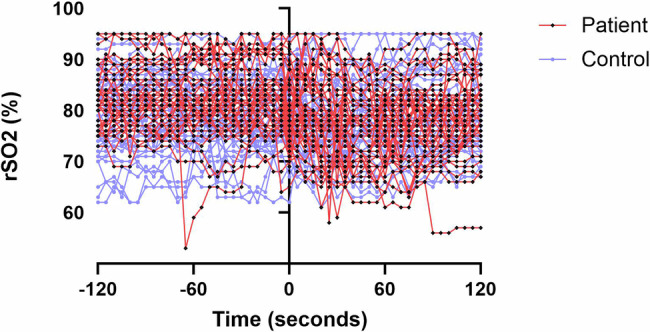
Diagram showing the changes in rScO_2_ during study.

the comparison of vital signs and the rScO_2_ values did not show any statistical significance between the groups (*p* > 0.05), the N-PASS score was significantly lower in the study group than in the control (1.36 ± 1.58 vs. 3.73 ± 3.26, *p* = 0.001, respectively) (Table 2). The rScO_2_ values measured in both groups during the study are shown in Fig. 3.

Additionally, the percent changes in rScO_2_, HR and SpO_2_ in both groups were compared according to time and groups using the Repeated-Measures ANOVA test in our study. It was found that the percent change in rScO_2_ values during and after the procedure did not differ between the groups (*p* = 0.209). However, it was observed that the percent change in rScO_2_ values in the study group decreased significantly compared to the percent change in rScO_2_ values in the control group (*p* = 0.033). Similarly, the percent change in HR and SpO_2_ was evaluated, it was seen that there was no significant difference according to both time and groups (*p* = 0.268 and *p* = 0.961, respectively).

However, it was determined that the percent changes in HR and SpO2 during and after the procedure decreased significantly compared to the baseline values in both groups (*p* = 0.011 and *p* = 0.001, respectively) (Table 3).

**Table 3:** The comparison of group-time interaction effects on the percent change of measurements during study period.

Parameters	Study Group [*n* = 45]		Control Group [*n* = 45]		Time	Grup	Grup-Time Interaction
	During procedure	After procedure	During procedure	After procedure	*p*	*p*	*p*
**rScO**_**2**_ **(%)**	−3.53 ± 5.65	−4.69 ± 6.55	−1.78 ± 6.13	−1.52 ± 5.65	0.429	0.033	0.209
**Heart rate (%)**	19.19 ± 12.35	12.75 ± 16.19	18.30 ± 14.89	15.74 ± 14.92	0.011	0.683	0.268
**SpO**_**2**_ **(%)**	−8.01 ± 6.30	−3.96 ± 6.19	−7.76 ± 6.05	−3.78 ± 6.14	0.001	0.843	0.961

Repeated-measures ANOVA.

Data are shown as mean ± SD.

rScO_2_ (%); Regional cerebral oxygen saturation.

SpO_2_ (%): Transcutaneous oxygen saturation.

According to the correlation between the percent change in rScO_2_ values and the percent change in HR and SpO_2_ during the procedure, there was a significantly positive correlation between rScO_2_ values and SpO_2_ (*r* = 0.331, *p* = 0.001), while no correlation was found between the percent change in rScO_2_ values and the percent change in HR (*r* = −0.29, *p* = 0.788). Additionally, when the correlation between rScO_2_ values and N-PASS scores was evaluated during the procedure, there was a significantly low negative correlation between rScO_2_ values and N-PASS scores (*r* = −0.236, *p* = 0.025)(Not shown).

## Discussion

This study was performed to assess the effect of administering breast milk on the responses of the brain’s prefrontal cortex using NIRS and on the clinical responses using N-PASS during a painful procedure for metabolic disease screening in healthy full-term newborns. Our findings indicate that while breast milk administration did not produce a statistically significant reduction in the cortical pain response compared with control group, breast milk administration infants had an appreciably lower response following heel prick.

Nonpharmacological analgesics have been previously studied by evaluating the cerebral activity elicited by clinical nociceptive stimulation. Our findings are consistent with those reported by Rioualen et al. who compared the two nonpharmacological strategies of breastfeeding and sucrose administration on the cortical responses to pain recorded using NIRS. They did not find any difference between sucrose administration (which is associated with holding and non-nutritive sucking) and breastfeeding on the activity measured using NIRS in the contralateral somatosensory cortex during venipuncture [2]. Additionally, Beken et al. investigated infant brain activity following venipuncture and found that dextrose caused greater changes in the cerebral blood volume in the left frontoparietal brain region but did not alter tissue oxygenation than administering sterile water, despite dextrose reducing behavioral scores [36] In another study, monitoring the cortical activity using NIRS during a heel stick after oral glucose administration revealed no activation of the parietal and frontal areas [20]. Ranger et al. evaluated the cerebral hemodynamic response of the therapeutic bed for procedural pain management in preterm infants and found no differences in the response patterns of the regional cerebral tissue oxygenation between infants receiving human touch-based treatment (control), facilitated tucking, and the therapeutic bed during heel lance [37]. Similarly, EEG recordings have been used to investigate the effects of sucrose on brain activity evoked by noxious stimuli. Slater et al. found that sucrose decreased behavioral responses to noxious stimuli without reducing the cortical response to pain [38]. Contrastingly, Bembich et al. evaluated the effects of breast milk and breastfeeding on pain-associated neonatal cortical activity using multichannel NIRS during a heel prick. They showed that administering breast milk resulted in bilateral activation of the motor and somatosensory areas during heel stick [22]. Moreover, breastfeeding was associated with extensive cortical activity involving the bilateral somatomotor and somatosensory cortices and the right posterior parietal cortex. They confirmed a possible role of multisensory somatic stimulation (tactile, proprioceptive, and thermal) in breastfeeding analgesia [39]. Fernandez et al. showed that sucrose attenuates the negative electroencephalographic (EEG) response to painful stimulus [40]. Olsson et al. investigated the effect of skin-to-skin contact on brain activity in preterm neonates who underwent venipuncture. They reported that kangaroo care alleviated pain as measured using NIRS in preterm infants during venipuncture, although they did not find a significant difference in the PIPP Revised score [41]. They emphasized that pain can be processed at the cortical level even when no behavioral signs exist. However, no significant differences were found in the NIRS measurements on the ipsilateral side in this study.

Clinical studies on neurophysiological methods applied to neonatal nonpharmacological analgesia have yielded conflicting results; in some studies, these methods were effective in the cortical pain response, whereas in others, they were not. These studies were not standardized in terms of patient numbers, gestational age, comparison group selection, procedure type, using different forms of nonpharmacological strategies (intervention types), pain assessment measures, and methods used, which may explain the conflicting results.

Some studies have evaluated changes in NIRS scores to discriminate pain in full-term and preterm neonates [18]. Near-infrared spectroscopy has demonstrated increased cortical activation in somatosensory areas of the brain in response to painful stimuli [19]. In our study, we found a significant decrease in the rScO_2_ values associated with the cortical pain response in both groups. Similarly, Nishino et al. reported a significant decrease in cerebral NIRS scores associated with cortical pain activity in newborn infants [42]. Pain appears to increase oxygen consumption in the prefrontal cortex. We consider that the heel prick is an extremely painful procedure, and NIRS may be used as an effective and safe method to monitor pain in newborns.

Behavioral, hormonal, metabolic, and other physiological changes occur in infants after exposure to noxious stimuli [9, 10]. In our study, the mean SpO_2_ values were lower and the mean heart rate values were higher during the heel prick procedure in both groups; however, the difference between the two groups was insignificant. Similar to our study, a meta-analysis comparing breastfeeding with other nonpharmacological interventions during vaccination in infants aged 1 to 12 months found no evidence that breastfeeding had an effect on physiological responses such as heart rate and SpO_2_ [26]. Unlike this study, our study involved populations of hospitalized infants undergoing heel-prick procedure for routine newborn screening. However, Upadhyay et al. found that the changes in heart rate and oxygen saturation were significantly lower in the expressed breast milk group (compared with our study, 5 ml was used) and returned to baseline values sooner than those in the distilled water group [29].

Additionally, when the effect of breast milk on the percent changes in in rScO_2_ values, HR and SpO_2_ during and after the procedure compared to baseline values was evaluated, it was found that there was no difference between the study and control groups in our study.

In our study, the N-PASS scores during and after the painful procedure were significantly lower in the study group than those in the control group. Our results are in accordance with those of a previous Cochrane systematic review exploring the analgesic effects of breastfeeding and breast milk supplementation, which indicated the superior analgesic effect of breastfeeding and supplemental breast milk compared to placebo, positioning, or no intervention [10]. These results suggest that breast milk significantly reduces behavioral responses to pain stimuli. Additionally, our investigation revealed conflicting data concerning cortical and behavioral responses to pain following supplemental breast milk intervention. Our results showed that 2 mL of supplemental breast milk did not affect prefrontal cortex activity. These hypotheses explain the following findings. First, breast milk administered using a syringe may not have the same efficacy as direct breastfeeding itself. Direct breastfeeding has a greater clinical analgesic effect than expressed breast milk alone, as it is a multisensory intervention that combines individual pain-reducing interventions, such as maternal closeness and olfactory and oral stimulation [10, 43]. However, direct breastfeeding with heel pricking has several problems. Some mothers find babies undergoing venipuncture disagreeable and prefer not to be present during the procedure; however, holding and skin-to-skin contact can influence pain perception [44]. Another hypothesis is that the amount of supplemental breast milk may be insufficient for the cerebral pain response, and the behavioral inhibition by the brainstem and not pain activation in the prefrontal cortex may influence the pain score.

Crying duration, as a primary pain measure, has been widely used in various studies [44]. In our study, we found that it was significantly lower in the study group than in the control group. Similarly, Upadhyay et al. showed that expressed breast milk significantly decreased the duration of crying after venipuncture [29]. Furthermore, Lin et al. demonstrated that neonates receiving either breast milk odor or breast milk odor and taste had a shorter cry duration during and after a heel-prick procedure compared to neonates in the control group [45]. However, other studies reported no differences in the pain scores or cry duration when breast milk odor and taste of the study group were compared with those of the control group during a painful procedure [46].

Some studies examining the relationship between NIRS responses and behavioral pain scores have found a significant correlation between NIRS responses and pain score [18]. However, in our study, a negative correlation existed between NIRS and N-PASS pain scores. These findings indicate that the cortical activity in response to pain may vary between the somatosensory and prefrontal areas in infants who have undergone painful procedures. Additionally, we found significantly positive correlation between the percentage changes in rScO_2_ and SpO_2_ during the procedure. This shows that the relationship between pain and oxygen consumption in the body is global.

This study presents multi-modal measurement of infant responses to painful stimuli and effect of breast milk on pain response. However, our study has several limitations, including the following: First, functional assessment with NIRS is limited to the cerebral cortex, and NIRS cannot match that of other neurobiology-based parameters (e.g., functional magnetic resonance imaging and electroencephalography). However, NIRS is relatively insensitive to movement artifacts and can be used at the bedside. Second, as our NIRS device was equipped with only one channel, we limited our cerebral monitoring to the prefrontal cortices; therefore, we could not fully evaluate the correlation between the other brain anatomical areas and the prefrontal areas in infants undergoing painful procedures. Therefore, using multichannel NIRS is more appropriate than using a single-channel probe. Third, our study included healthy full-term neonates who underwent a unique and painful procedure. Therefore, these results may not apply to sick or preterm infants undergoing multiple acute, painful, or stressful procedures. Fourth, the sample size was calculated according to the N-PASS score and not the NIRS measurements. Finally, as this was a single-center study, further multicenter and large-scale studies are required to confirm these findings.

## Conclusion

This study makes a novel contribution to the literature by the heel prick is an extremely painful procedure, and administering 2 mL of breast milk is insufficient for reducing cortical pain. Therefore, further effective treatment methods are required to reduce pain during minor procedures in newborns. Additional research to understand the pain relief mechanisms, whether there is a dose-dependent effect of expressed breast milk, and the specific impact of each nonpharmacological method is required.

## Data Availability

The datasets generated and/or analysed during the current study are available from the corresponding author on reasonable request.
